# Acidic Fibroblast Growth Factor Promotes Endothelial Progenitor Cells Function via Akt/FOXO3a Pathway

**DOI:** 10.1371/journal.pone.0129665

**Published:** 2015-06-10

**Authors:** Liya Huang, Fei Wang, Yuqiang Wang, Qing Cao, Tiantian Sang, Fang Liu, Shuyan Chen

**Affiliations:** Department of Geriatrics, Xinhua Hospital affiliated to Shanghai Jiaotong University School of Medicine, Shanghai, China; Centro Cardiologico Monzino, ITALY

## Abstract

Acidic fibroblast growth factor (FGF1) has been suggested to enhance the functional activities of endothelial progenitor cells (EPCs). The Forkhead homeobox type O transcription factors (FOXOs), a key substrate of the survival kinase Akt, play important roles in regulation of various cellular processes. We previously have shown that FOXO3a is the main subtype of FOXOs expressed in EPCs. Here, we aim to determine whether FGF1 promotes EPC function through Akt/FOXO3a pathway. Human peripheral blood derived EPCs were transduced with adenoviral vectors either expressing a non-phosphorylable, constitutively active triple mutant of FOXO3a (Ad-TM-FOXO3a) or a GFP control (Ad-GFP). FGF1 treatment improved functional activities of Ad-GFP transduced EPCs, including cell viability, proliferation, antiapoptosis, migration and tube formation, whereas these beneficial effects disappeared by Akt inhibitor pretreatment. Moreover, EPC function was declined by Ad-TM-FOXO3a transduction and failed to be attenuated even with FGF1 treatment. FGF1 upregulated phosphorylation levels of Akt and FOXO3a in Ad-GFP transduced EPCs, which were repressed by Akt inhibitor pretreatment. However, FGF1 failed to recover Ad-TM-FOXO3a transduced EPCs from dysfunction. These data indicate that FGF1 promoting EPC function is at least in part mediated through Akt/FOXO3a pathway. Our study may provide novel ideas for enhancing EPC angiogenic ability and optimizing EPC transplantation therapy in the future.

## Introduction

Coronary atherosclerotic heart disease is one of the most common types of organ lesions caused by atherosclerosis. Vascular endothelial cell injury and dysfunction are the initiators of atherosclerosis. Endothelial progenitor cells (EPCs) have been shown to be involved in postnatal neovascularization and vascular repair [1–3]. In recent years, EPC transplantation for treatment of ischemic heart disease, has been tested in pilot clinical trials and achieved some encouraging results [4–6]. However, studies [7–8] have also shown that the number and functional activities of EPCs are impaired in patients with classical cardiovascular risk factors, potentially leading to a limitation for autologous transplantation of EPCs. Therefore, maintaining or promoting EPC function may be the premise to ensure therapeutic effects of EPCs.

Acidic fibroblast growth factor (FGF1) is one of the most important angiogenic factors that exerts its effects through binding to the surface receptors of endothelial cells and initiates intracellular signal transduction pathways, with resultant modification of cell function such as cell proliferation and migration [9]. Our previous study [10] has demonstrated the functional enhancement of EPC viability, migration and tube formation by FGF1, however the molecular mechanism(s) whereby FGF1 promotes EPC function has not been completely understood.

The Phosphatidylinositol-3 kinase (PI3K)/Akt signaling pathway mediates cell survival for various growth factors [11]. The Forkhead homeobox type O transcription factors (FOXOs), one of the key substrates of the survival kinase Akt, play important roles in regulating cell growth [12–14]. Akt phosphorylates FOXOs and leads to cytoplasmic translocation and transcriptional inhibition, resulting in promotion of cell proliferation and suppression of cell apoptosis [15]. Our recent studies have confirmed that human cord blood derived EPCs predominantly expressed FOXO3a and its overexpression led to increased EPC apoptosis [16] as well as decreased EPC proliferation [17]. Some other studies have indicated that apoptosis [18] and migration [19] of endothelial cells are under the regulation of PI3K/Akt/FOXO3a pathway. However, there are few studies about the regulation of EPC function by FGF1 and its potential molecular mechanism. Therefore, the present study aims to determine whether FGF1 promotes EPC function via Akt/FOXO3a signaling pathway.

## Materials and Methods

This work was approved by Medical Ethics Committee Affiliated Xinhua Hospital of School of medicine Shanghai Jiaotong University (Approval No. XHEC-D-2013-007). Written informed consent from the donor of the human peripheral blood was obtained.

### EPC cultivation and identification

Human peripheral blood mononuclear cells (PBMCs) were separated and collected by density gradient centrifugation with Histopaque-1077 (Sigma) and seeded on fibronectin (Gibco) precoated 6-well culture plates. The EGM-2 MV BulletKit (Lonza Clonetics) complete medium was used and changed every three days. The adherent cells were passaged at approximately 80% confluence after two weeks of culture. Third to fourth passage cells were used for the subsequent experiments.

For EPC identification, cells were immunofluorescently stained using antibodies against endothelial markers CD34 (Epitomics) and VEGFR-2 (R&D Systems) as well as stem/progenitor marker CD133 (Origene). Additionally, cells were identified by flow cytometry after staining with the above three antibodies. Dual staining for acetylated low density lipoprotein (Dil-AcLDL, Molecular Probes) uptake and lectin from Ulex europaeus (FITC-UEA-I, Sigma) binding was also used for EPC confirmation [20].

### Adenoviral construction and transduction

The triple mutant form of FOXO3a, in which mutations occurred in three conservative Akt phosphorylation sites (Thr32, Ser253 and Ser315), could not be phosphorylated by Akt and maintain its transcriptional activity in the nucleus [18]. The adenoviral vector expressing a non-phosphorylable, constitutively active triple mutant of FOXO3a (Ad-TM-FOXO3a) was constructed as previously described [16]. In brief, the human FOXO3a triple mutant sequence cDNA (purchased from Genecopoeia USA) in which its conservative three Akt phosphorylation sites were replaced by alanine residues was subcloned into a shuttle vector pShuttle-CMV-EGFP. The recombinant shuttle plasmid was transformed into Escherichia coli bacteria and then recombined with the adenoviral backbone plasmid pAdeno. The recombinant adenoviral plasmid was transfected into 293K cells for packaging and amplification of adenovirus. The adenoviral vector expressing only the GFP transgene (Ad-GFP) used as the control was constructed by the same system.

For EPC transduction, cells at approximately 90% confluence were transduced with the recombinant adenoviral vectors at a multiplicity of infection (MOI) of 40. After two hours of incubation, cells were washed and fresh medium was added. GFP green fluorescent protein expression and cell morphology were observed to determine the transduction efficiency under fluorescence microscope (Leica DMI3000B). Cells were harvested at 48 hours of transduction for the subsequent experiments as described below.

### CCK-8 assay

To determine the optimal concentration and effect time of FGF1 (Gibco) and Akt inhibitor [1L6-Hydroxymethyl-chiro-inositol-2-(R)-2-O-methyl-3-O-octadecyl-sn-glycerocarbonate,Merck] on EPC viability, a Cell Counting Kit-8 (CCK-8, Dojindo) assay was used. 5x10^3^ cells in 100μl Medium were seeded in each well of 96-well plates in triplicate. The Medium used for CCK-8 assay contained EBM-2 basal medium with 5% FBS but without supplementary growth factors. After treatment with different concentrations (5, 25, 50ng/ml) of FGF1 and incubation for different time durations (12, 24, 48 hours), 10μl CCK-8 solution was added to each well for four hours, and light absorbance at 450nm was measured using an ELISA plate reader (BIO-TEK ELX800). Furthermore, EPCs were pretreated with different concentrations (2.5, 5, 10μM) of Akt inhibitor for one hour, and then FGF1 was added and incubated for the determined concentration and effect time.

To determine whether FGF1 regulates EPC function via Akt/FOXO3a signaling pathway, cells were divided into five groups and all the following experiments were performed with the five groups. That is, Ad-GFP (Ad-GFP transduced EPCs), Ad-GFP+FGF1 (Ad-GFP transduced EPCs with FGF1 treatment), Ad-GFP+Akt inhibitor+FGF1 (Ad-GFP transduced EPCs with Akt inhibitor pretreatment before adding with FGF1), Ad-TM-FOXO3a (Ad-TM-FOXO3a transduced EPCs) and Ad-TM-FOXO3a+FGF1 (Ad-TM-FOXO3a transduced EPCs with FGF1 treatment), respectively. Then, CCK-8 assay was performed. The CCK-8 assay and the following experiments were all independently repeated three times.

### Flow cytometry analysis for cell proliferation and cell apoptosis

In order to assess the changes in cell proliferation, FxCycle PI/RNase Staining Solution (Invitrogen) was used according to the manufacturer's instructions. The Medium used for cell proliferation assay contained EBM-2 basal medium with 5% FBS but without supplementary growth factors. 3x10^5^ cells in 2ml Medium were seeded per well in 6-well plates in triplicate with or without FGF1 and Akt inhibitor. After treatment, adherent and non-adherent cells were collected and fixed with ice-cold 70% ethanol at -20°C overnight. After washing steps, FxCycle PI/RNase Staining Solution was added and incubated for 30 minutes at room temperature in the dark. Cells were analyzed with flow cytometry (BD FACSCanto II).

Cell apoptosis was detected by dual staining with APC Annexin V and PI (Molecular Probes). The Medium used for cell apoptosis assay contained EBM-2 basal medium with 5% FBS but without supplementary growth factors. 1x10^5^ cells in 1ml Medium were seeded per well in 24-well plates in triplicate with or without FGF1 and Akt inhibitor. Adherent and non-adherent cells were harvested and resuspended in Annexin-binding buffer, stained with APC Annexin V and PI for 15 minutes at room temperature protected from light, and then analyzed with flow cytometry as soon as possible.

### Migration assay

Transwell chamber (diameter 6.5mm used for 24-well plate, aperture 8μm; Corning Costar) was used to observe the cell migration. The Medium used for cell migration assay contained EBM-2 basal medium with 5% FBS but without supplementary growth factors. 5x10^4^ cells in 100μl Medium were added to the upper chamber, and 600μl Medium with or without FGF1 and Akt inhibitor were added to the lower chamber. Then, the non-transmigrated cells were scraped and the transmigrated cells were fixed with 4% paraformaldehyde and stained with 0.1% hexamethylpararosaniline. Three randomly microscopic fields were selected to enumerate the cells.

### Tube formation assay

Matrigel basement membrane matrix (BD Biosciences) was used to evaluate the tube formation ability of the cells. The Medium used for tube formation assay contained EBM-2 basal medium with 5% FBS but without supplementary growth factors. In brief, Matrigel was thawed at 4°C and added to 24-well plates, followed by incubation at 37°C for 30 minutes. Then, 1x10^5^ cells in 1 ml Medium were seeded per well on gel-formed Matrigel with or without FGF1 and Akt inhibitor. After overnight of incubation, three randomly microscopic fields were selected to observe the capillary-like structures and calculate the number of closed network units.

### Western blotting

Total cellular proteins were extracted and their concentrations were measured. To confirm whether FGF1 promotes EPC function associated with Akt/FOXO3a phosphorylation, equal quantity of protein samples from the following three groups, Ad-GFP, Ad-GFP+FGF1 and Ad-GFP+Akt inhibitor+FGF1, was loaded onto SDS-PAGE and blotted onto polyvinylidene fluoride (PVDF) membrane (Millipore, Billerica, MA). Furthermore, to determine whether FOXO3a dephosphorylation abolishes the beneficial effects of FGF1 on EPC function, equal quantity of protein samples from the following three groups, Ad-GFP, Ad-TM-FOXO3a and Ad-TM-FOXO3a+FGF1, was loaded onto SDS-PAGE and blotted onto PVDF membrane (Millipore, Billerica, MA). Western blotting was performed by the use of primary antibodies against Akt (1: 1000, Santa Cruz Biotechnology), phospho-Akt [p-Akt(Ser473) 1: 1000, Santa Cruz Biotechnology], FOXO3a (1: 1000, Cell Signaling Technology), and phospho-FOXO3a [p-FOXO3a(Ser253) 1: 1000, Cell Signaling Technology], followed by incubation with horseradish peroxidase (HRP)-conjugated secondary antibodies (Jackson Laboratories) at appropriate dilutions and detected by ECL detection reagents (Millipore, Billerica, MA). Anti-β-actin antibody (1: 1000, Cell Signaling Technology) was used as loading control. The levels of protein expression were analyzed by chemiluminescence imaging (BIO-RAD ChemiDoc XRS).

### Statistical analysis

Data are expressed as mean ± SD. Comparisons between groups were analyzed by two-tailed Student's *t* test or ANOVA when appropriate. A probability value of < 0.05 (*P* < 0.05) was considered statistically significant. All analyses were performed with SPSS 17.0 software.

## Results

### Identification of EPCs

**Fig 1:**
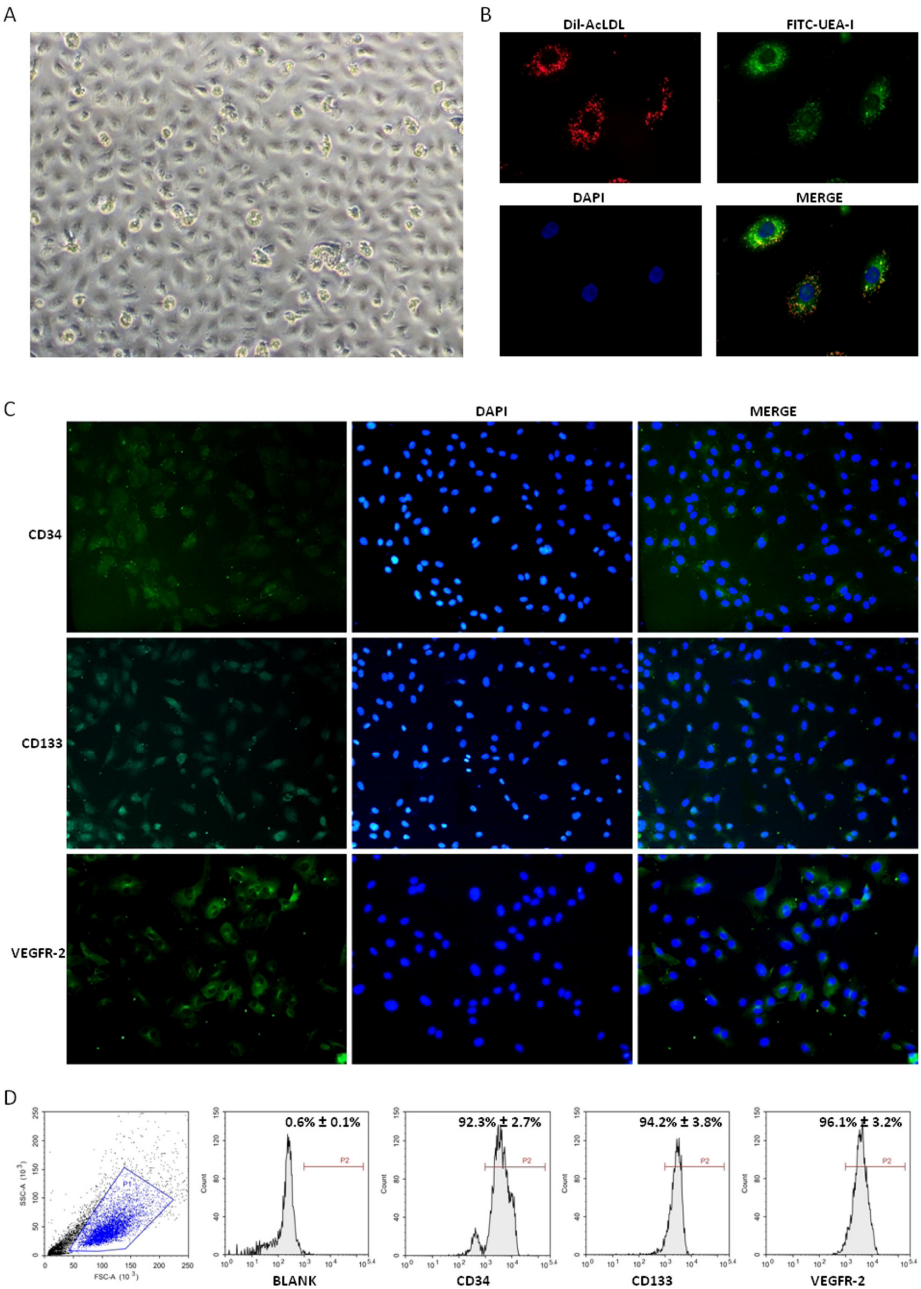
Identification of EPCs from human PBMCs. (A) Monolayer of first passage EPCs with a cobblestone morphology (magnification×100). (B) Immunofluorescence staining of EPCs double positive for Dil-AcLDL and FITC-UEA-I (magnification×400). (C) Immunofluorescence staining of EPCs positive for CD34, CD133 and VEGFR-2 (magnification×200). (D) A representative FSC/SSC plot and the expression of EPC markers (CD34, CD133 and VEGFR-2) analyzed with flow cytometry. Data are mean ± SD, n = 3.

Under the present culture conditions, the adherent cells from human PBMCs showed a cobblestone morphology after two weeks of culture (Fig 1A), and they were double positive staining for Dil-AcLDL and FITC-UEA-I (Fig 1B). Immunofluorescence staining showed that the cultured cells expressed endothelial markers CD34 and VEGFR-2 as well as stem/progenitor marker CD133 (Fig 1C). Flow cytometry analysis revealed that the positive expression rate of CD34 was 92.3% ± 2.7%, and 96.1% ± 3.2% for VEGFR-2, as well as 94.2% ± 3.8% for CD133 (Fig 1D). All these are considered as the important characteristics of EPCs.

### Adenoviral transduction and transgene expression in EPCs

**Fig 2:**
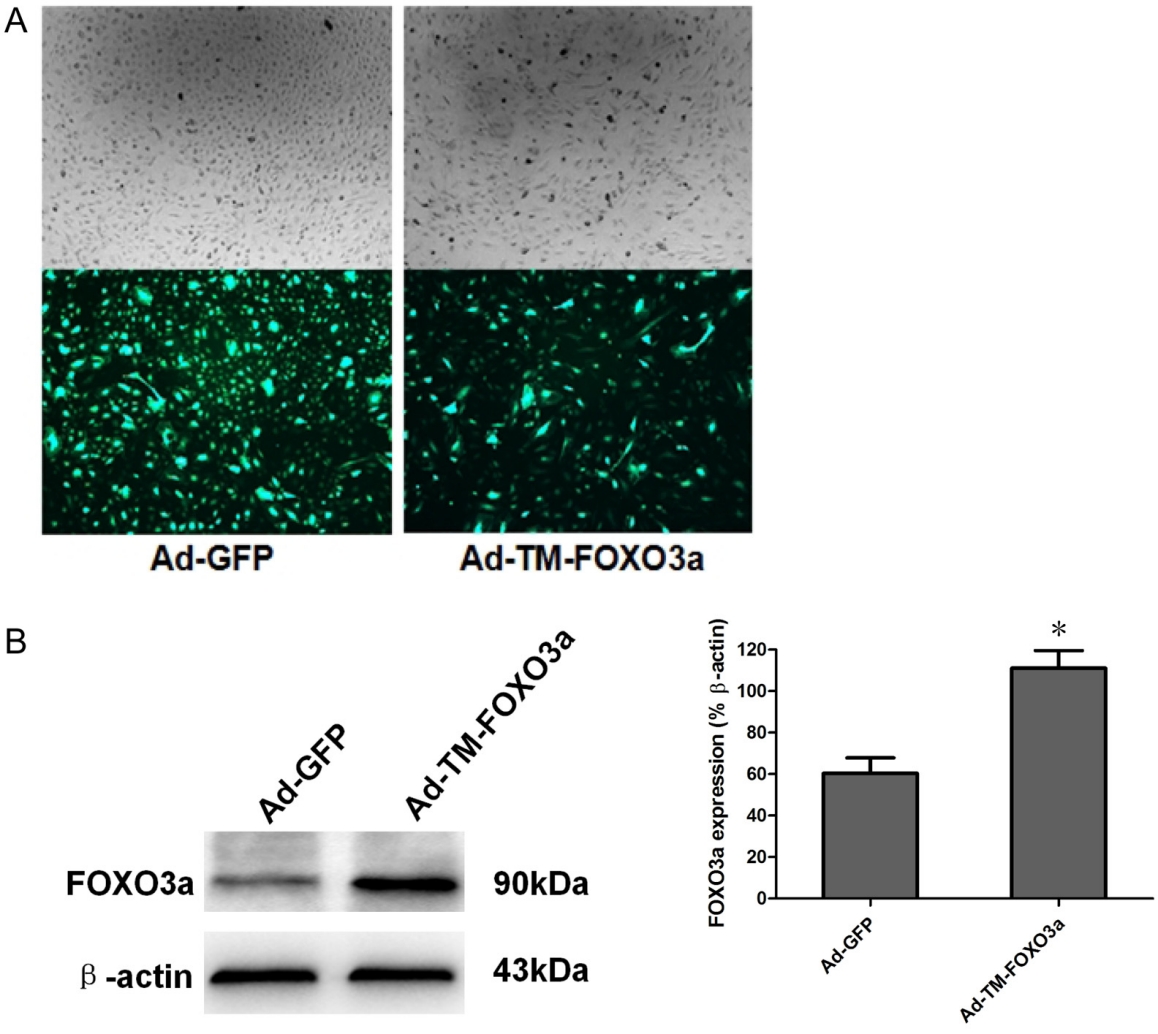
Adenoviral transduction and transgene expression in EPCs. (A) Morphology of EPCs at 48 hours of transduction with Ad-GFP under normal light (Upper Left) and view of the same field under green fluorescent light (Below Left); morphology of EPCs at 48 hours of transduction with Ad-TM-FOXO3a under normal light (Upper Right) and view of the same field under green fluorescent light (Below Right) (magnification×50). (B) At 48 hours of transduction with Ad-GFP or Ad-TM-FOXO3a, cells were lysed and FOXO3a protein level was detected by western blotting. β-actin was used as loading control. FOXO3a expression was quantified by densitometric analysis. The ratios for FOXO3a/β-actin are shown. Data are mean ± SD, n = 3, two-tailed Student’s *t* test was used for statistical analysis, *P<0.05 vs Ad-GFP.

After 48 hours of transduction, the green fluorescent protein GFP was observed in EPCs, which confirmed the high transduction efficiency of adenoviral vectors (Fig 2A). Western blotting confirmed the FOXO3a protein overexpression in Ad-TM-FOXO3a transduced EPCs (Fig 2B). There were no morphological changes of EPCs after Ad-GFP transduction (Fig 2A: Upper Left and Below Left). However, Ad-TM-FOXO3a transduced EPCs showed poor growth characteristics: some cells adhered incompletely and the intercellular gaps increased, as well as a decrease in the number of adherent cells compared with Ad-GFP transduced EPCs (Fig 2A: Upper Right and Below Right).

### EPC viability assay

**Fig 3:**
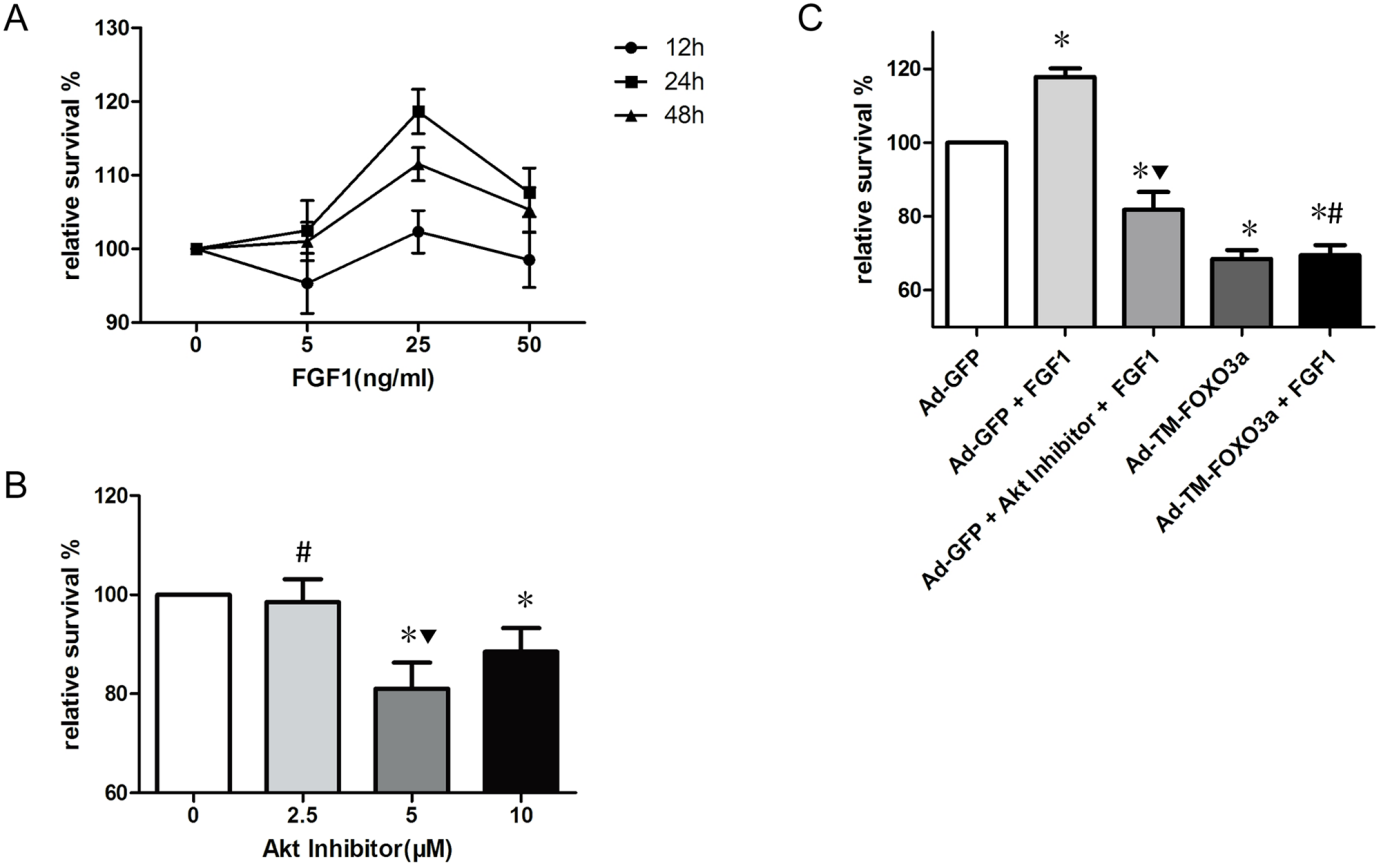
FGF1 increased EPC viability associated with Akt and FOXO3a signals by CCK-8 assay. (A) EPCs were treated with different concentrations of FGF1 and incubated for different time durations. Data are mean ± SD, n = 3, Two-way ANOVA was used for statistical analysis. (B) EPCs were pretreated with different concentrations of Akt inhibitor for one hour, followed by adding with 25ng/ml FGF1 for 24 hours. Data are mean ± SD, n = 3, One-way ANOVA was used for statistical analysis, *P<0.05 vs 0μM; ▼P<0.05 vs 10μM;#P = NS vs 0μM. (C) EPCs were divided into five groups, Ad-GFP (Ad-GFP transduced EPCs), Ad-GFP+FGF1 (Ad-GFP transduced EPCs with FGF1 treatment), Ad-GFP+Akt inhibitor+FGF1 (Ad-GFP transduced EPCs with Akt inhibitor pretreatment before adding with FGF1), Ad-TM-FOXO3a (Ad-TM-FOXO3a transduced EPCs) and Ad-TM-FOXO3a+FGF1 (Ad-TM-FOXO3a transduced EPCs with FGF1 treatment), respectively. Data are mean ± SD, n = 3, One-way ANOVA was used for statistical analysis, *P<0.05 vs Ad-GFP; ▼*P*<0.05 vs Ad-GFP+FGF1; #*P* = NS vs Ad-TM-FOXO3a.

By CCK-8 assay, the optimal concentration and effect time of FGF1 on EPC viability was 25ng/ml for 24 hours (Fig 3A) and the optimal concentration of Akt inhibitor was 5μM (Fig 3B), which were used and for all the following experiments. In addition, CCK-8 assay was also used to evaluate the viabilities of EPCs transduced with the indicated vectors as well as treated with or without FGF1 and Akt inhibitor. Results suggested that FGF1 promoted EPC viability but Akt inhibitor pretreatment abolished the beneficial effect of FGF1, Ad-TM-FOXO3a transduction inhibited EPC viability compared with the Ad-GFP group and FGF1 failed to attenuate EPC dysfunction by FOXO3a overexpression (Fig 3C).

### EPC proliferation and apoptosis assay

**Fig 4:**
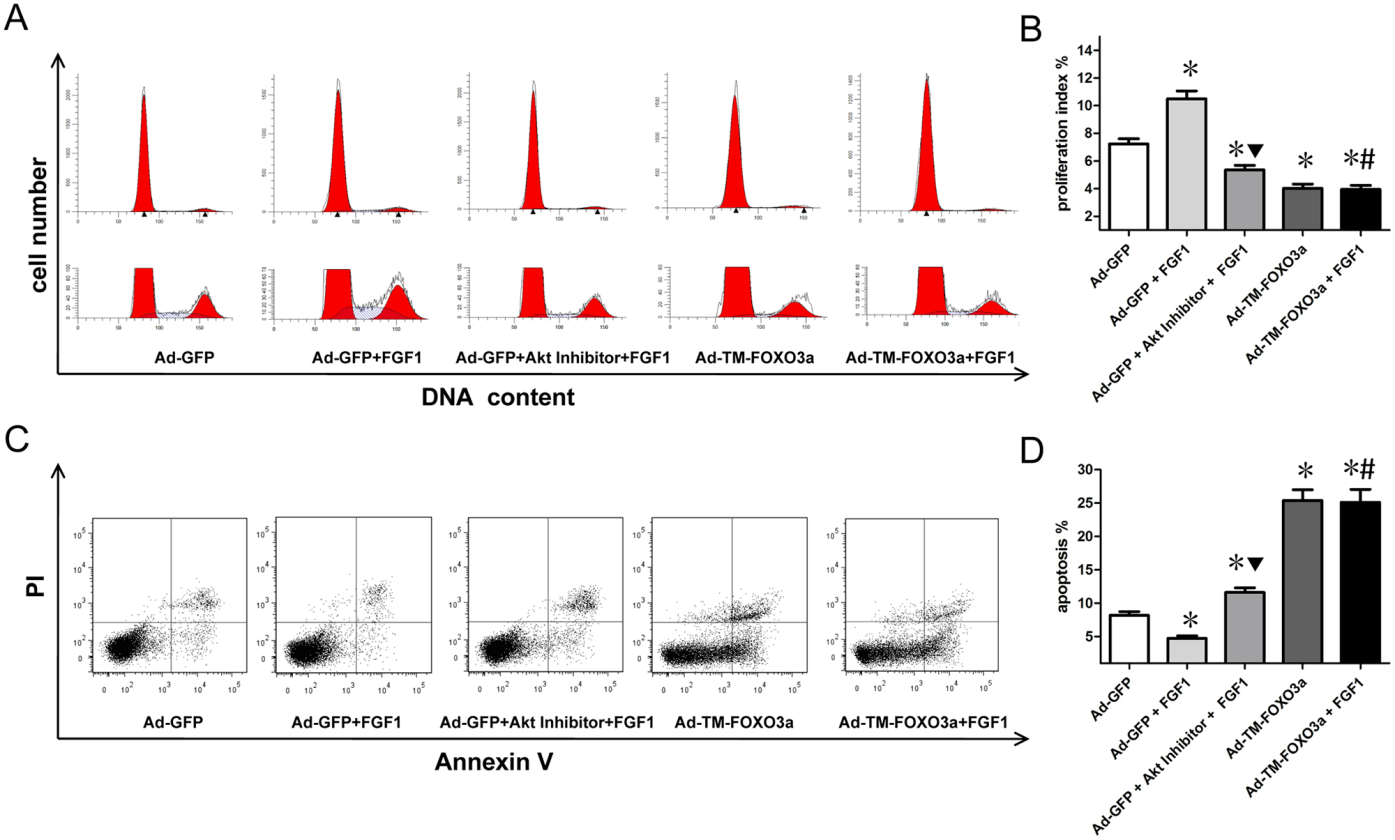
FGF1 enhanced EPC proliferation and protected EPCs from apoptosis associated with Akt and FOXO3a signals. (A) Cell proliferation was detected by PI/RNase staining and analyzed with flow cytometry. (B) Proliferation Index (PI) was used to evaluate EPC proliferation. PI = (S+G2M)/(G0/1+S+G2M). Data are mean ± SD, n = 3, One-way ANOVA was used for statistical analysis, *P<0.05 vs Ad-GFP; ▼*P*<0.05 vs Ad-GFP+FGF1; #*P* = NS vs Ad-TM-FOXO3a. (C) Cell apoptosis was detected by Annexin V and PI dual staining and analyzed with flow cytometry. (D) The percentage of apoptotic cells with Annexin V positive expression was used to assess EPC apoptosis. Data are mean ± SD, n = 3, One-way ANOVA was used for statistical analysis, *P<0.05 vs Ad-GFP; ▼*P*<0.05 vs Ad-GFP+FGF1; #*P* = NS vs Ad-TM-FOXO3a.

Proliferation Index (PI) was used to evaluate EPC proliferation (Fig 4B). PI = (S+G2M)/(G0/1+S+G2M). Flow cytometry analysis (Fig 4A and 4B) showed that FGF1 enhanced EPC proliferative activity while Akt inhibitor pretreatment abolished this effect of FGF1. FOXO3a overexpression decreased EPC proliferative activity, and FGF1 failed to attenuate EPC dysfunction by FOXO3a overexpression.

Cell apoptosis assay (Fig 4C and 4D) with Annexin V and PI dual staining revealed that FGF1 protected EPCs from apoptosis while pretreatment with Akt inhibitor abolished this effect. FOXO3a overexpression increased EPC apoptosis that was unable to be attenuated by FGF1 treatment.

### EPC migration and tube formation assay

**Fig 5:**
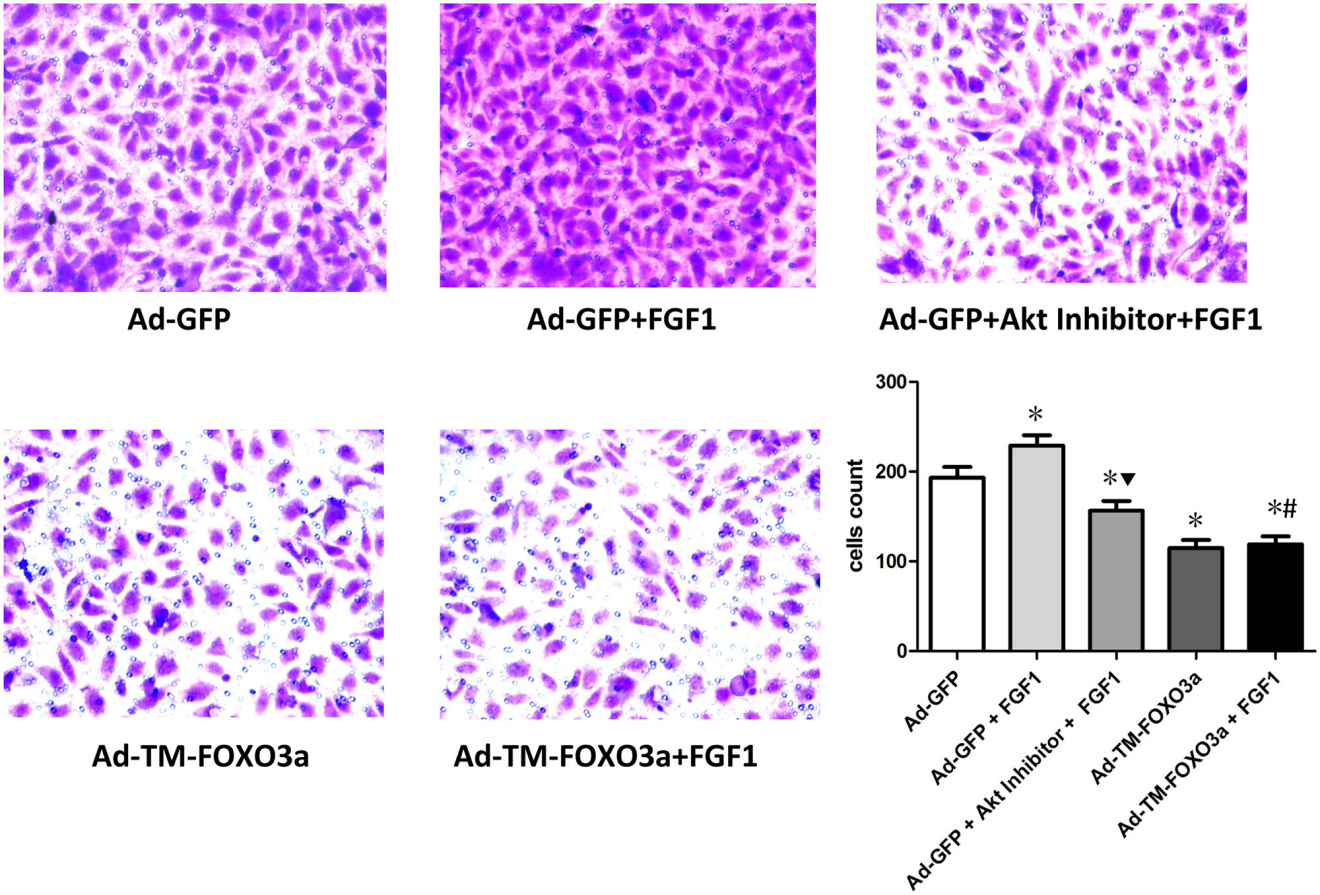
FGF1 improved EPC migration associated with Akt and FOXO3a signals. The representative photomicrographs from Transwell assay were shown (magnification×200). Three randomly microscopic fields were selected to enumerate the cells. Data are mean ± SD, n = 3, One-way ANOVA was used for statistical analysis, *P<0.05 vs Ad-GFP; ▼*P*<0.05 vs Ad-GFP+FGF1; #*P* = NS vs Ad-TM-FOXO3a.

Transwell assay was used to assess EPC migration. As illustrated in Fig 5, FGF1 improved EPC migration while Akt inhibitor pretreatment abolished this effect. Ad-TM-FOXO3a transduction inhibited EPC migration that was unable to be recovered by FGF1 treatment.

**Fig 6:**
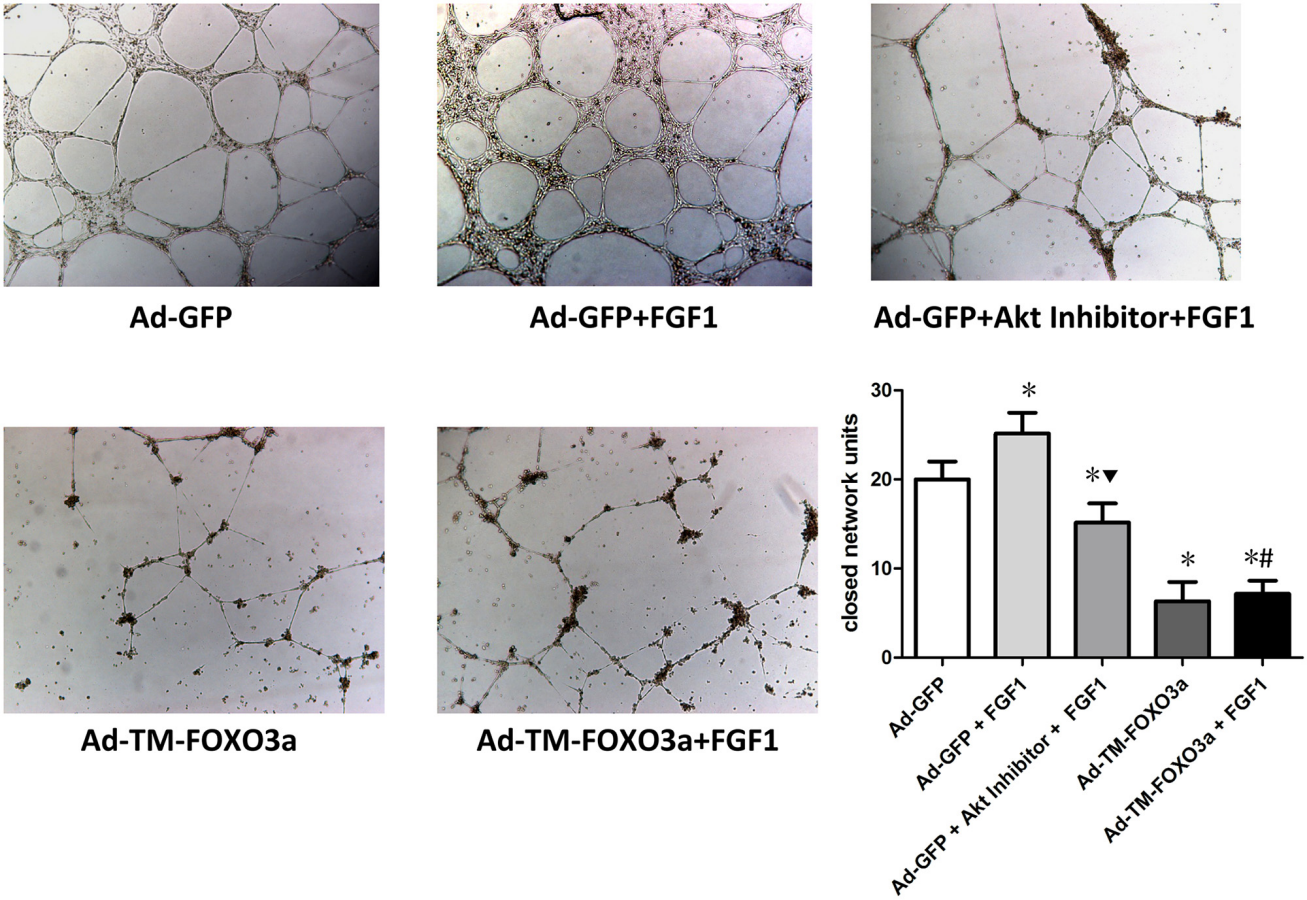
FGF1 augmented EPC tube formation ability associated with Akt and FOXO3a signals. The representative photomicrographs from Matrigel assay were shown (magnification×40). Three randomly microscopic fields were selected to observe the capillary-like structures and calculate the number of closed network units. Data are mean ± SD, n = 3, One-way ANOVA was used for statistical analysis, *P<0.05 vs Ad-GFP; ▼*P*<0.05 vs Ad-GFP+FGF1; #*P* = NS vs Ad-TM-FOXO3a.

We examined the tube forming ability of EPCs using Matrigel assay. As illustrated in Fig 6, FGF1 augmented EPC tube formation ablity but its beneficial effect was abolished by Akt inhibitor pretreatment. FOXO3a overexpression reduced EPC tube formation, and FGF1 failed to make EPC dysfunction recovery by FOXO3a overexpression.

### FGF1 promotes EPC function through Akt/FOXO3a pathway

**Fig 7:**
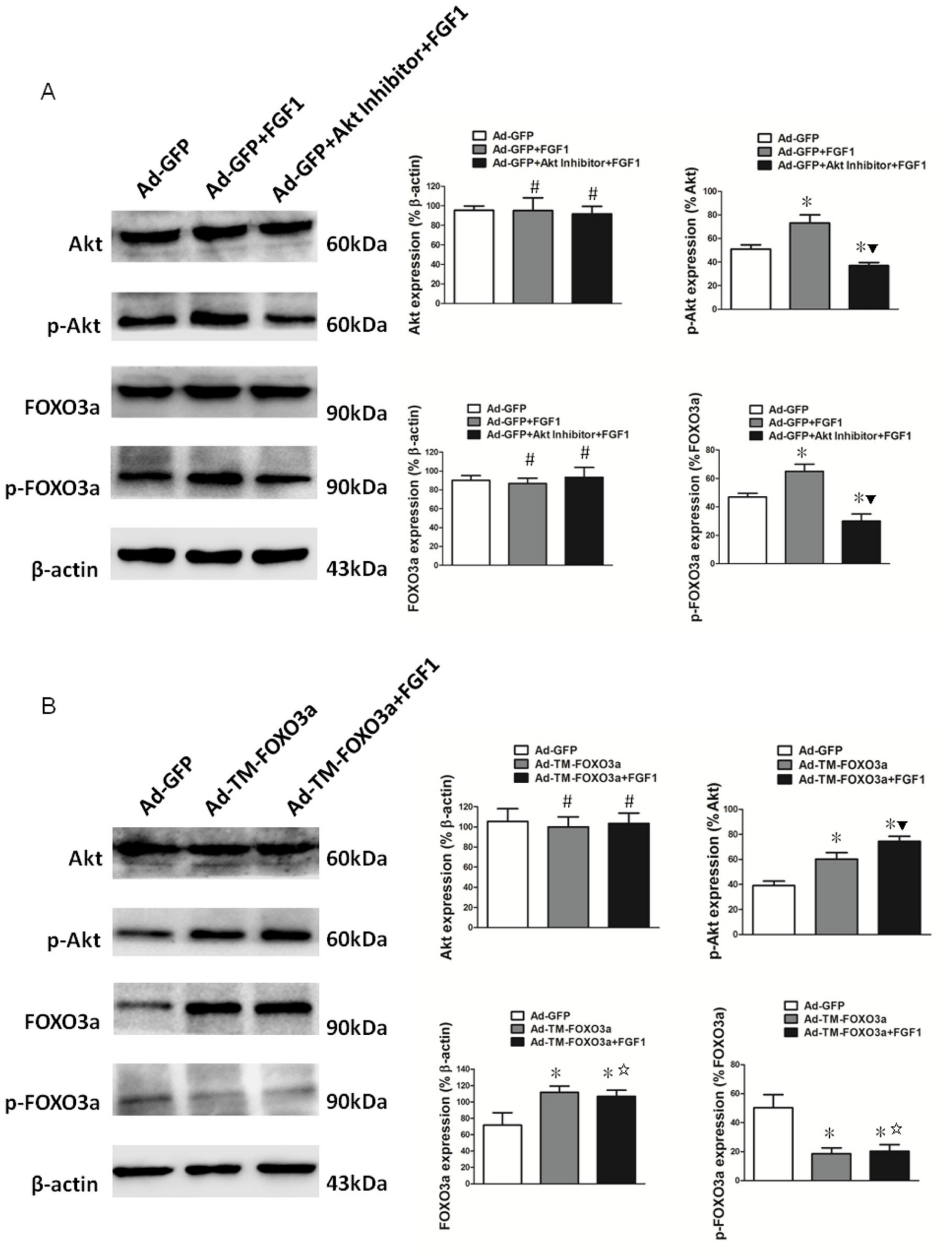
FGF1 promoted EPC function through Akt/FOXO3a pathway. (A) After treatment with or without FGF1 and Akt inhibitor, Ad-GFP transduced EPCs were lysed and protein levels of Akt, p-Akt, FOXO3a and p-FOXO3a were detected by western blotting. β-actin was used as loading control. Protein expression was quantified by densitometric analysis. Data shown are the mean ± SD of the ratios for Akt/β-actin, p-Akt/Akt, FOXO3a/β-actin and p-FOXO3a/FOXO3a. One-way ANOVA was used for statistical analysis, #*P* = NS vs Ad-GFP; *P<0.05 vs Ad-GFP; ▼*P*<0.05 vs Ad-GFP+FGF1. (B) EPCs were transduced with the indicated vectors and treated with or without FGF1. Cells were lysed and protein levels of Akt, p-Akt, FOXO3a and p-FOXO3a were detected by western blotting. β-actin was used as loading control. Protein expression was quantified by densitometric analysis. Data shown are the mean ± SD of the ratios for Akt/β-actin, p-Akt/Akt, FOXO3a/β-actin and p-FOXO3a/FOXO3a. One-way ANOVA was used for statistical analysis, #*P* = NS vs Ad-GFP; *P<0.05 vs Ad-GFP; ▼*P*<0.05 vs Ad-TM-FOXO3a; ☆*P* = NS vs Ad-TM-FOXO3a.

Western blotting was performed to compare the protein expression levels of Akt, p-Akt, FOXO3a and p-FOXO3a so as to determine whether FGF1 promotes EPC function through Akt/FOXO3a pathway. Ad-GFP transduced EPCs with FGF1 treatment displayed increased p-Akt and p-FOXO3a expression and showed decreased p-Akt and p-FOXO3a expression by Akt inhibitor pretreatment (Fig 7A). In Ad-TM-FOXO3a transduced EPCs, there was higher FOXO3a but lower p-FOXO3a expression compared with the Ad-GFP group, however, FOXO3a as well as p-FOXO3a expression showed no changes with or without FGF1 treatment (Fig 7B). In addition, the Ad-TM-FOXO3a group displayed enhanced p-Akt expression compared with the Ad-GFP group whether or not treated with FGF1 (Fig 7B).

## Discussion

In this study, EPCs were cultured from human peripheral blood and transduced with adenoviral vectors either expressing a non-phosphorylable, constitutively active triple mutant of FOXO3a or a GFP control. Results showed that FGF1 improved EPC function while Akt inhibitor reversed these beneficial effects, and FOXO3a overexpression decreased EPC function that was unable to be attenuated by FGF1 treatment. Collectively, FGF1 promoting EPC function is at least in part mediated through Akt/FOXO3a pathway.

Studies have shown that two types of EPC are developed based on their surface antigens and growth characteristics under in vitro culture conditions [21–23]. One type is called early EPCs, also known as circulating angiogenic cells, coexpress endothelial and macrophage/monocyte antigens and almost have no proliferative potential. The other is called late EPCs, also named as outgrowth endothelial cells, coexpress endothelial and stem/progenitor markers and exhibit a high capacity of proliferation. According to the characteristics of different EPCs, the human peripheral blood derived EPCs obtained in the present study are well consistent with the late EPCs. It is known that EPC function is associated with various cardiovascular risk factors, such as hypertension, diabetes, hypercholesterolemia and aging, leading to a limitation for EPC transplantation [7–8, 24]. Thus, improving EPC function has become an important research focus.

FGF1, a prototypic member of the FGF family that contains at least 20 polypeptides with angiogenic properties, is a potent mitogen for a variety of cell types, such as endothelial cells and vascular smooth muscle cells [10]. FGF1 is confirmed by its ability to influence key steps of angiogenesis, including endothelial cell detachment, migration, proliferation and differentiation [25]. FGFs interact with FGFR1, which is the main FGF receptor expressed in endothelial cells [9] as well as EPCs [26] and its activation induces several signal transduction pathways, leading to multiple biological changes such as cell proliferation and migration. Moreover, it has been reported that FGFs can upregulate VEGF expression in endothelial cells [27], which is well known to promote the function of both endothelial cells and EPCs. Our previous study [10] has shown that FGF1 overexpression improved the functional properties of EPCs, however the molecular mechanism(s) by which FGF1 promotes EPC function remains to be determined.

We previously have shown that FOXO3a is the main subtype of FOXOs expressed in EPCs [16]. FOXO3a is widely expressed in human tissues and it is a highly conserved molecule in the cascade of signal transduction pathways [28]. Studies have shown that upregulation of FOXO3a expression activates negative cell cycle regulation factors such as p27 and p21, or inhibits positive cell cycle regulation factors such as CDK4, CDK6 and cyclin D1, leading to cell cycle arrest and cell proliferation inhibition [29–31]. In addition, upregulation of FOXO3a expression increases FasL and Bim expression which play important roles in cell apoptosis [32–33]. Our previous study has also shown that FOXO3a overexpression causes EPC dysfunction through transcriptional regulation of its downstream target genes such as p27, CDK2, cyclin D1, PCNA and Bim [16–17]. It is known that PI3K/Akt signaling pathway mediates cell survival for various growth factors and FOXO3a is one of the key substrates of Akt [11, 34]. Furthermore, decreased activity and downregulated expression of FOXO3a occurs in the presence of growth signals, such as epidermal growth factor receptor (EGFR), insulin, insulin-like growth factor (IGF) and some cytokines [35–36]. Phosphorylation/dephosphorylation plays important roles in the regulation of FOXO3a activity. The amino acid sequence of FOXO3a contains three highly conserved Akt phosphorylation sites (Thr32, Ser253 and Ser315) [18]. Akt phosphorylates FOXO3a and leads to cytoplasmic translocation and transcriptional inhibition, resulting in promotion of cell proliferation and suppression of cell apoptosis [15]. Therefore, the favorable effects of FGF1 on EPC function have been supposed to be partly mediated through phosphorylation of Akt and FOXO3a. FGF1 phosphorylates and activates Akt which in turn promotes phosphorylation and inactivation of FOXO3a, leading to transcriptional regulation of its downstream target genes, and eventually improves EPC function. Interestingly, Ad-TM-FOXO3a transduced EPCs showed significantly enhanced p-Akt expression compared with the Ad-GFP group whether or not treated with FGF1, which was consistent with the results of some studies that FOXO3a overexpression could negative feedback activate PI3K/Akt pathway [37–38]. Therefore, it is likely that FOXO3a overexpression might promote activation of Akt which in turn increases FOXO3a phosphorylation. However, Ad-TM-FOXO3a transduction significantly reduced FOXO3a phosphorylation in EPCs since the mutant FOXO3a could not be phosphorylated by Akt in these cells.

In summary, we have demonstrated that FGF1 comprehensively improves EPC function and mechanistically these favorable effects are at least in part mediated through Akt/FOXO3a pathway. Our study may provide novel ideas for enhancing EPC angiogenic ability and optimizing EPC transplantation therapy in the future.
